# The impact of modifying photosystem antenna size on canopy photosynthetic efficiency—Development of a new canopy photosynthesis model scaling from metabolism to canopy level processes

**DOI:** 10.1111/pce.13041

**Published:** 2017-09-21

**Authors:** Qingfeng Song, Yu Wang, Mingnan Qu, Donald R. Ort, Xin‐Guang Zhu

**Affiliations:** ^1^ Chinese Academy of Sciences Center for Excellence in Molecular Plant Sciences, Institute of Plant Physiology and Ecology, Shanghai Institutes for Biological Sciences Chinese Academy of Sciences Shanghai 200032 China; ^2^ State Key Laboratory of Hybrid Rice and CAS‐MPG Partner Institute for Computational Biology, Shanghai Institutes for Biological Sciences Chinese Academy of Sciences Shanghai 200031 China; ^3^ Institute of Genomic Biology University of Illinois at Urbana Champaign Champaign IL 61801 USA; ^4^ Global Change and Photosynthesis Research Unit United States Department of Agriculture, Agricultural Research Service Champaign IL 61801 USA

**Keywords:** antenna size, biomass, canopy, photosynthesis model, photosynthetic efficiency

## Abstract

Canopy photosynthesis (A_c_) describes photosynthesis of an entire crop field and the daily and seasonal integrals of A_c_ positively correlate with daily and seasonal biomass production. Much effort in crop breeding has focused on improving canopy architecture and hence light distribution inside the canopy. Here, we develop a new integrated canopy photosynthesis model including canopy architecture, a ray tracing algorithm, and C_3_ photosynthetic metabolism to explore the option of manipulating leaf chlorophyll concentration ([Chl]) for greater A_c_ and nitrogen use efficiency (NUE). Model simulation results show that (a) efficiency of photosystem II increased when [Chl] was decreased by decreasing antenna size and (b) the light received by leaves at the bottom layers increased when [Chl] throughout the canopy was decreased. Furthermore, the modelling revealed a modest ~3% increase in A_c_ and an ~14% in NUE was accompanied when [Chl] reduced by 60%. However, if the leaf nitrogen conserved by this decrease in leaf [Chl] were to be optimally allocated to other components of photosynthesis, both A_c_ and NUE can be increased by over 30%. Optimizing [Chl] coupled with strategic reinvestment of conserved nitrogen is shown to have the potential to support substantial increases in A_c_, biomass production, and crop yields.

## Abstract 2

Decreasing leaf chlorophyll concentration ([Chl]) by reducing photosystem antenna size can not only improve the availability of light at lower layers of a canopy but also increase the proportion of light absorbed by leaf for photochemistry. Coupling decreasing leaf chlorophyll concentration with strategic reinvestment of conserved nitrogen can lead to substantial increases in canopy photosynthetic CO_2_ uptake and hence crop yield potential.

## 1 INTRODUCTION

Canopy photosynthesis (A_c_) describes photosynthesis of both top and bottom layer leaves. Because the seasonal integration of A_c_ is highly correlated with biomass production (Wells, Meredith, & Williford, 1986; Wells, Schulze, Ashley, Boerma, & Brown, 1982; Zelitch, 1982), many studies have focused on how to maximize A_c_ (e.g., Field, 1983; Hirose & Werger, 1987; Shiratsuchi, Yamagishi, & Ishii, 2006). In the canopy, upper layer leaves usually absorb more light than its saturation level and the excess light energy is dissipated mainly through heat dissipation, but lower layer leaves are usually limited by available light. Improving the distribution of light inside a canopy can increase light use efficiency and hence increase canopy photosynthesis. Increasing light penetration into bottom layers of a canopy can be realized through manipulating canopy structure, which has been applied in crop breeding. For instance, erect leaves were selected during rice breeding, which increases light penetration into bottom layers of a canopy (Peng, Khush, & Cassman, 1994). Besides canopy architecture, decreasing leaf chlorophyll concentration has also been suggested as another potential method to improve light distribution and hence light use efficiency inside crop canopies (Ort, Zhu, & Melis, 2011; Zhu, Long, & Ort, 2010). However, previous theoretical studies used models in which the microclimatic condition inside a canopy were dramatically simplified. In particular, the light environment inside the canopy was mainly divided into only sunlit and shaded (Norman, 1980). Our theoretical analysis has shown that such a simplification leads to up to 17% bias in the estimated total canopy photosynthetic CO_2_ uptake rate (Zhu, Song, & Ort, 2012). In addition, two major advances in recent years now make possible development of a new generation of dynamic systems model of canopy photosynthesis, where both the light environment inside the canopy and also the detailed photosynthetic processes are integrated (Zhu, Wang, Ort, & Long, 2013).

The first major development is the tool to predict the light environment inside a canopy (Song, Zhang, & Zhu, 2013). Light inside a canopy is highly heterogeneous both spatially and temporally (Pearcy, 1990). The leaves in the lower layers usually have low light levels; however, these low light levels are sporadically interrupted by high light sunflecks (Pearcy, 1990), which make up to a large proportion of the incident solar energy on lower canopy leaves. A lot of previous efforts to model canopy photosynthesis, including the classical big leaf model (Running & Coughlan, 1988; Sellers, Berry, Collatz, Field, & Hall, 1992; Thornley & Johnson, 1990), sunlit/shaded model (Dai, Dickinson, & Wang, 2004; DePury & Farquhar, 1997; Wang & Leuning, 1998), and multilayer model (DeWit, 1965; Lemon, Stewart, & Shawcroft, 1971; Norman, 1979), do not fully consider the high level of spatial and temporal heterogeneities of light inside the canopy. Zhu and colleagues used a reverse ray tracing algorithm combined with a simplified canopy architecture to predict the spatial and temporal heterogeneity inside an idealistic canopy (Zhu, Ort, Whitmarsh, & Long, 2004). Using this model, the potential impact of formation and relaxation of photoprotection inside a canopy was explored, which led to the discovery that the natural slow recovery from photoprotected state could lead to up to 30% loss of A_c_ (Zhu et al., 2004). Recently, algorithms to reconstruct three‐dimensional canopy architecture and algorithms for forward ray tracing were developed, enabling a more accurate prediction of light environment of a canopy and allowing for user‐defined canopy architecture parameters (Song et al., 2013).

A comprehensive dynamic systems model of leaf photosynthesis, which incorporates description of the detailed processes including both the electron transfer processes and the dynamics of carbon metabolism, has also been developed recently (Zhu et al., 2013). This model, in comparison to earlier steady state biochemical photosynthesis model (Farquhar, Caemmerer, & Von, & Berry J.A., 1980), can predict the dynamic changes of photosynthesis under varying light and CO_2_ levels. This improved model, known as e‐photosynthesis, is also able to predict the potential impacts of manipulation of different components to leaf photosynthetic efficiency. By combining with evolutionary algorithms, we are now able to explore the optimal nitrogen distribution into different enzymes of photosynthetic carbon metabolism to maximize photosynthesis. Combining this advanced dynamic leaf photosynthesis model with modelling of the heterogeneous light environment within canopies enables prediction of dynamic changes of canopy photosynthesis in any canopy of defined architecture.

The e‐photosynthesis model, in which each photosynthetic reaction and process is explicitly represented, also enables the study of the nitrogen investment to maximize photosynthesis (Zhu et al., 2013). Because light varies widely in different layers within canopies, there is photo‐acclimation of leaves to irradiance that changes as canopy grows. It is well known that leaves under higher growth light tend towards higher nitrogen content per leaf area as indicated by the observed decline in nitrogen content with light levels inside the canopy (Evans & Poorter, 2001; Field, 1983; Hikosaka, 2005). In addition, nitrogen distribution among photosynthetic enzymes within leaves are different under different growth irradiance (Evans, 1993a; Evans, 1993b; Evans & Poorter, 2001; Hikosaka & Terashima, 1995; Niinemets, Kull, & Tenhunen, 1998). For example, under higher growth light, more nitrogen is partitioned to Rubisco and electron transport chain components, as compared to low growth light where nitrogen investment shifts towards light harvesting (Evans, 1989). Combining a realistic light environment inside a canopy with the e‐photosynthesis model offers the opportunity to investigate the optimal nitrogen distribution among photosynthetic enzymes within those leaves.

In this study, we have assembled an integrated canopy photosynthesis model by combining canopy architecture model (Song et al., 2013), ray tracing algorithm (Song et al., 2013), photo‐acclimation model (Hikosaka & Terashima, 1995; Kull & Kruijt, 1999; Moreau et al., 2012), and dynamic systems model of C_3_ leaf photosynthesis (Zhu et al., 2013). Using this model, we have systematically evaluated the effects of reducing leaf chlorophyll concentration to light and nitrogen use efficiencies of a rice canopy.

## 2 MATERIAL AND METHODS

### 2.1 Plant materials and experiments

Rice cultivar 9522 (Oryza sativa L. japonica) was planted in the experimental station in Shanghai (Latitude 31°N) in 2012 with a planting density 25 × 20 cm^2^ (20 plants/m^2^). Canopy architectural features and the physiological parameters were collected in the booting stage (August 23, 235 DOY). The leaf reflectance and transmittance were measured using integrating sphere and spectrometer (Ocean Optics, Dunedin, Florida, USA). The leaf reflectance (*r*) and transmittance (*t*) were then calculated according to the following equations (Equations (1)–(2)), where the *r*_*n*_ is reflectance of wave length *n* and *I*_*n*_ is light intensity at wave length *n*.

(1) $$r=\sum_{n=400}^{700}r_{n}\cdot I_{n}/\sum_{n=400}^{700}I_{n},$$

(2) $$t=\sum_{n=400}^{700}t_{n}\cdot I_{n}/\sum_{n=400}^{700}I_{n}.$$

**Figure 1:**
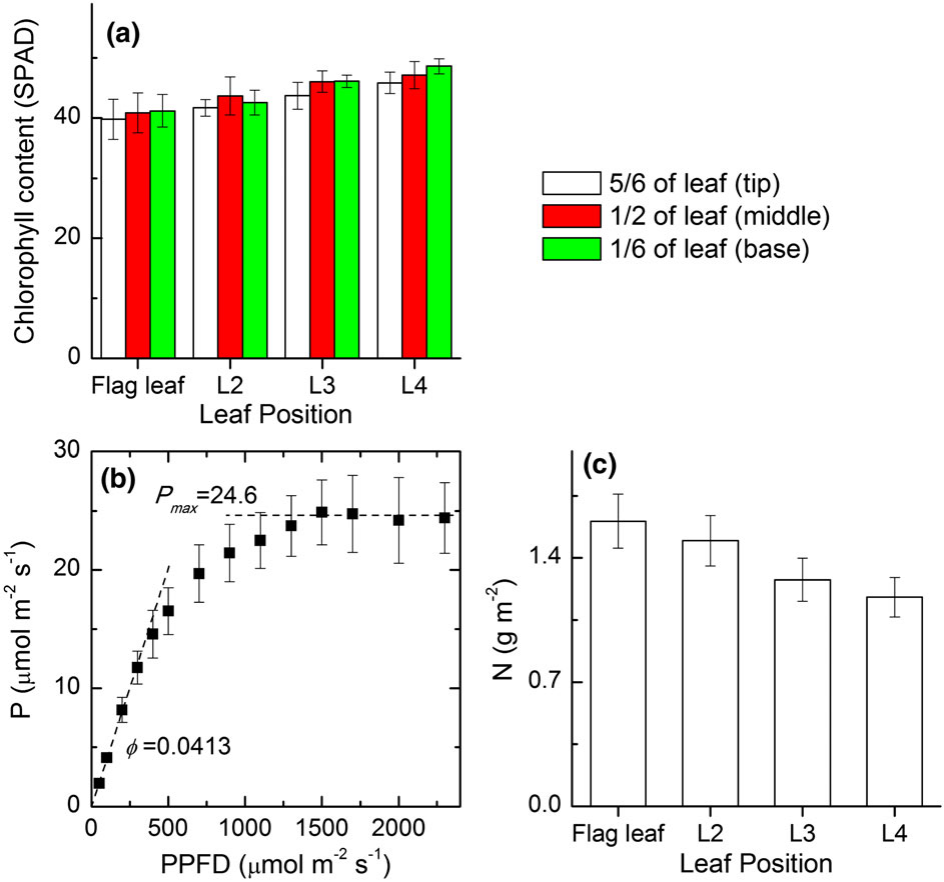
Leaf physiological parameters used during the simulations. (a) Leaf chlorophyll contents represented by the SPAD readings of different segments of the flag leaf, the second leaf, the third leaf, and the fourth leaf. (b) Light response curves of photosynthesis (A‐Q curve) of the flag leaf, the maximal photosynthetic CO_2_ uptake rate under ambient CO_2_ and saturate photosynthetic photon flux density (PPFD; *P*_max_) and the initial slope of the A‐Q curve (*ϕ*)fitted with a nonrectangular hyperbola model. (c) Leaf nitrogen contents for different leaf positions. Flag leaf nitrogen content was measured and other leaves was predicted based on model (Equations (3)–(4); mean ± std, *n* = 5)

We measured the SPAD values using a chlorophyll metre SPAD‐502Plus (Konica Minolta, Japan) for different leaf segments, that is, the leaf base at 1/6 of the leaf length, leaf middle segment at 1/2 of the leaf length, and leaf tip at 5/6 of the length, of the flag leaf, the second leaf, the third leaf, and the fourth leaf (Figure 1a). Chlorophylls at these different segments were also extracted with 95% ethanol to measure concentrations using spectrophotometer following Arnon (1949). The chlorophyll concentrations and the corresponding SPAD readings were used to derive a relationship between chlorophyll concentration and single‐photon avalanche diode (SPAD) reading (Equation S2). Photosynthesis was measured with the gas exchange method using LI‐6400XT (LI‐COR, Lincoln, Nebraska, USA). Light response curves of flag leaves were measured under a CO_2_ concentration of 400 ppm and the photosynthetic photon flux density (PPFD) was changed stepwise from 2,400 to 50 μmol·m^−2^·s^−1^. (Figure 1b). The *P*_max_ (the maximal light saturated photosynthesis under ambient CO_2_ concentration) and *ϕ* (the initial slope of light response curve) were fitted with a nonrectangular hyperbola model (Thornley, 2002). Leaf nitrogen content of the flag leaf was determined using a Hanon Instrument Model K9840 Kjeldahl Distillation Unit (Hanon, Shandong, China). The nitrogen contents of other leaves were predicted with the model in derived in (Moreau et al., 2012; Figure 1c).

### 2.2 Canopy model and ray tracing algorithm

A 3D canopy structure model representing nine rice plants was constructed using *mCanopy* (PICB, Shanghai) with parameters collected from rice plants described above using the methods described in Song et al. (2013). A ray tracing algorithm was applied to simulate PPFD distribution in this canopy using the software *fastTracer* developed in Song et al. (2013), software available upon request from authors. The light environments inside the canopy at four rice growing regions of China, that is, Harbin (Latitude 45°N), Beijing (Latitude 40°N), Shanghai (Latitude 31°N), and Sanya (Latitude 18°N), were predicted by combining the canopy architecture model with *fastTracer* (Song et al., 2013). The simulation was conducted for Aug 23 (235 DOY) with a time interval of 0.5 hr. Leaf transmittance (*t*) and reflectance (*r*) were calculated with leaf chlorophyll concentrations for all leaves based on the equations fitted with measurement data (Equations S3–S4, data in Table S1). *t* and *r* were then used to parameterize the ray tracing programme to simulate PPFD distribution inside a canopy.

### 2.3 Nitrogen distribution in canopy

A model that describes the relationship between vertical nitrogen distribution and light distribution within a canopy (Moreau et al., 2012) was used to estimate the nitrogen profile in different canopies. In the model (Equations (3) and (4)), *N*_*LA*_ is nitrogen per leaf area, $N_{\mathrm{LA}}^{\mathrm{fl}}$is the *N*_*LA*_ in flag leaf, and *n*_*b*_ (g N m^−2^ leaf lamina) is the basal leaf nitrogen concentration. *I*_*l*_ is PPFD incident on the flag leaf and *I*_*lfl*_ is *I*_*l*_ at the middle of a flag leaf layer as *N*_*LA*_ of a layer was related to *I*_*l*_ */I*_*lfl*_ (Milroy, 2001) and *b* is equal to the ratio of extinction coefficient of nitrogen and light in canopy. In this study, we calculated *b* based on green leaf area index (*GAI*) using the equation used in Moreau et al., 2012.

(3) $$N_{\mathrm{LA}}=\left(N_{\mathrm{LA}}^{\mathrm{fl}}-n_{b}\right)\cdot {\left(\frac{I_{l}}{I_{l_{\mathrm{fl}}}}\right)}^{b}+n_{b},$$

(4) $$b=\beta \cdot {\mathrm{GAI}}^{-\alpha }.$$

### 2.4 Enzymes and proteins concentration calculated with photo‐acclimation model

A photo‐acclimation model for nitrogen partitioning among major photosynthetic proteins in a leaf was developed to link e‐photosynthesis (Zhu et al., 2013) and leaf nitrogen concentration. The e‐photosynthesis model can predict the amount of leaf absorbed PPFD used for photochemistry, heat dissipation, and fluorescence emission (Zhu et al., 2013). The photo‐acclimation model assumes that leaves optimize the distribution of nitrogen among photosynthetic components for maximizing the daily carbon uptake per leaf area (Hikosaka & Terashima, 1995). The photosynthesis enzymes and proteins are divided into several groups, (a) Rubisco, (b) enzymes in Calvin‐Benson cycle except Rubisco (CE), (c) electron transport chain and F_1_F_0_ ATPase (ETCF), (d) photosystem II (PSII), (e) photosystem I (PSI), (f) light‐harvesting complex II (LHCII), and (g) light‐harvesting complex I (LHCI). The parameters of light response curves of leaf photosynthesis, maximal photosynthesis rate *P*_max_ and quantum yield *ϕ*, are limited by different components of photosynthesis. *P*_max_ is limited by Rubisco, CE, and ETCF, and *ϕ* is limited by *PSII*. To build the photo‐acclimation model, first, the relationship between concentrations of major components and leaf photosynthesis parameters, *P*_max_ and *ϕ*, were generated using e‐photosynthesis model (Equations (5)–(8)); second, the diurnal PPFD absorbed by a leaf was simulated using *fastTracer* software for 5 days to generate an average diurnal growth PPFD curve of 5 days; third, the molecular weights and nitrogen contents of these groups were calculated, and at a given nitrogen content, the relationship between *P*_max_ and *ϕ* was generated; and finally, for the simulated averaged diurnal growth PPFD, a range of *P*_max_ and corresponding *ϕ* were used to calculate daily carbon uptake and the optimal *P*_*max*_ and *ϕ* for maximal daily carbon uptake were selected, then the concentrations of Rubisco, CE, ETCF, and PSII were calculated based on their relationships to *P*_max_ and *ϕ*.

(5) $$P_{\max}=a_{1}\times \left[\text{Rubisco}\right]+b_{1},$$

(6) $$P_{\max}=a_{2}\times \left[\mathrm{CE}\right]+b_{2},$$

(7) $$P_{\max}=a_{3}\times \left[\text{ETCF}\right]+b_{3},$$

(8) $$\phi =a_{4}\times \left[\text{PSII}\right]+b_{4}.$$

### 2.5 Leaf photosynthetic CO_2_ uptake calculated with e‐photosynthesis model

The e‐photosynthesis model (Zhu et al., 2013) was parameterized with the enzymes concentrations (*c*) and catalytic numbers (*k*_*cat*_). First, the enzymes and proteins in photosynthesis were divided into seven groups as described above. Within each group, the ratios among enzymes were set constant (Table S3) and the concentrations of those groups were calculated from leaf nitrogen content and environmental light according to photo‐acclimation model described above. The *k*_*cat*_ of all enzymes are for typical C_3_ plants as used in Zhu et al., 2013; Table S3). The *V*_max_ of all enzymes were then calculated by equation (Equation (9)).

(9) $$V_{\max}=k_{\mathrm{cat}}\cdot c.$$

### 2.6 Chlorophylls in antennas of PSII and PSI

The antenna of both PSII and PSI is divided into core antenna containing 37 and 95 chlorophyll molecules, respectively, according to Glick & Melis (1988) and peripheral antenna consist of n_1_ units of light‐harvesting complexes (LHCs) with 14 chlorophyll molecules in each LHC unit (Liu et al., 2004). The total chlorophyll concentration ([Chl]) was calculated by Equation (10) and when assume the ratio of [PSI]/[PSII] as 1.4, the total [Chl] was calculated by Equation (11).

(10) $$\left[\mathrm{Chl}\right]=\left[\text{PSII}\right]\cdot \left(37+14n_{1}\right)+\left[\mathrm{PSI}\right]\cdot \left(95+14n_{1}\right),$$

(11) $$\left[\mathrm{Chl}\right]=\left[\text{PSII}\right]\cdot \left(170+33.6n_{1}\right).$$

### 2.7 Integrated canopy photosynthesis model

**Figure 2:**
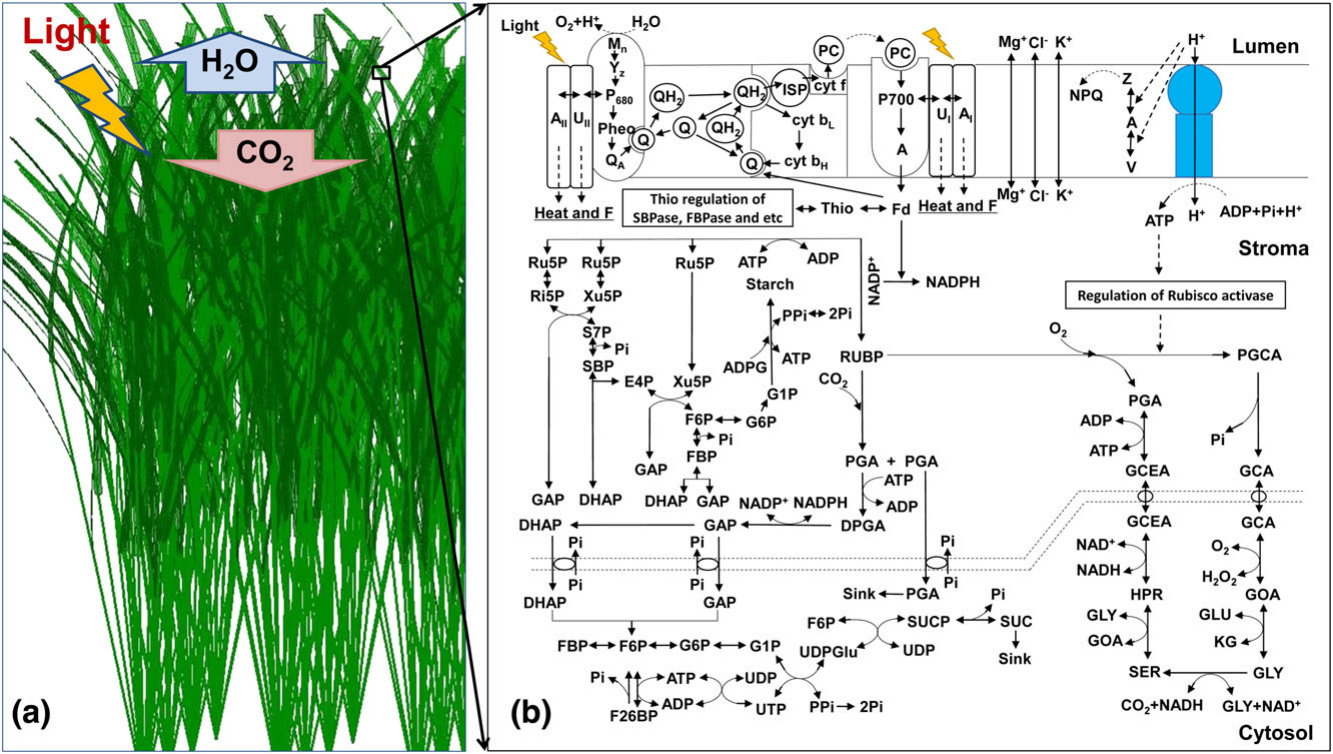
A diagram showing the integrated canopy model of rice that combines 3D canopy structure, ray tracing algorithm, and e‐photosynthesis model. The 3D canopy model was constructed based on rice canopy structural parameters. The ray tracing algorithm follows Song et al. (2013) and predicts photosynthetic photon flux density (PPFD) of all leaves in the canopy. The PPFD was used as input of the e‐photosynthesis to calculate the leaf photosynthetic rate. Canopy photosynthetic CO_2_ uptake rate was calculated as the integral of photosynthetic CO_2_ uptake rates of all leaves. The diagram of photosynthesis is adapted from Zhu et al. (2013) with permission

With the above individual modules, an integrated canopy photosynthesis model (Figure 2) scaling from metabolism to canopies was assembled. First, the 3D canopy architectural model and ray tracing algorithm were used to simulate the diurnal growth PPFD for leaves in canopy. Second, the PPFD distribution inside the canopy was used to estimate the distribution of nitrogen per leaf area in canopy. Third, using the photo‐acclimation model with input of the simulated PPFD and nitrogen per leaf area, enzyme concentrations were calculated. Finally, with the e‐photoysnthesis model parameterized by above enzymes concentrations and catalytic numbers, the CO_2_ assimilation rates for leaves in the canopy was calculated. Finally, canopy photosynthetic CO_2_ uptake rate was calculated as the sum of the product of leaf assimilation rate multiplied by facet area for all facets in a canopy.

### 2.8 Simulation scenarios

In the model, two vertical chlorophyll concentration ([Chl]) distributions inside canopies were (a) uniform [Chl] distribution and (b) measured [Chl] distribution. Two strategies to modify nitrogen contents were (a) changing LHC without affecting other photosynthetic components and (b) changing LHC and other photosynthesis components simultaneously by maintaining the total nitrogen in photosynthesis components constant. Four scenarios were generated based on different combinations of [Chl] distribution and nitrogen content manipulations (Table 1). The measured canopy architectural and leaf chlorophyll content of the rice cultivar 9522 were used as default canopy. The canopies with different chlorophyll contents were generated through modifying antenna size of photosystems.

**Table 1:** The four scenarios of changing chlorophyll concentration used for the model simulations

	Uniform [Chl] distribution	Measured [Chl] distribution
Change LHC only	Scenario 1	Scenario 2
Constant N per leaf area	Scenario 3	Scenario 4

### 2.9 Simulation of canopy photosynthesis under different leaf chlorophyll concentrations

The integrated canopy photosynthesis model was used to simulate four scenarios of modifying leaf chlorophyll concentrations. The chlorophyll concentration was assumed as following either a uniform distribution or measured distribution in a canopy. For uniform chlorophyll distribution scenarios, the measured canopy of the rice cultivar 9522 with averaged chlorophyll concentration of 494.7 μmol m^−2^ was used as default model and a series of models with 0.4, 0.6, 0.8, 1.0, and 1.2 times chlorophyll concentrations of default model were generated. For measured chlorophyll concentration scenarios, the canopy with measured leaf chlorophyll concentration was used and the averaged chlorophyll concentration was 494.7 μmol m^−2^. This model was used as default model and a series of models with 0.4, 0.6, 0.8, 1.0 and 1.2 times chlorophyll concentrations of default model were generated.

The leaf chlorophyll was changed by changing antenna size of photosystems, when leaf chlorophyll concentration was determined in the model, the concentrations of LHCII and LHCI were changed according to Equation (11). The leaf nitrogen per leaf area was also changed when changing LHCII and LHCI. In the four scenarios, two approaches were applied to change LHC. For the first approach, the amount of LHCII and LHCI was changed without changing the concentration of other enzymes. For the second approach, the amount of nitrogen changed (Δ*N*) was calculated when changing LHCII and LHCI, and then the Δ*N* was distributed to all the other photosynthesis enzymes proportionally to these enzymes concentrations. To test the generality of the effects of decreasing antenna size on A_c_, we simulated the A_c_ under different chlorophyll concentrations for canopy of different leaf angles, leaf area index, plant height, and locations.

## 3 RESULTS

### 3.1 Efficiencies of photosystems for two options of changing leaf chlorophyll concentrations

**Figure 3:**
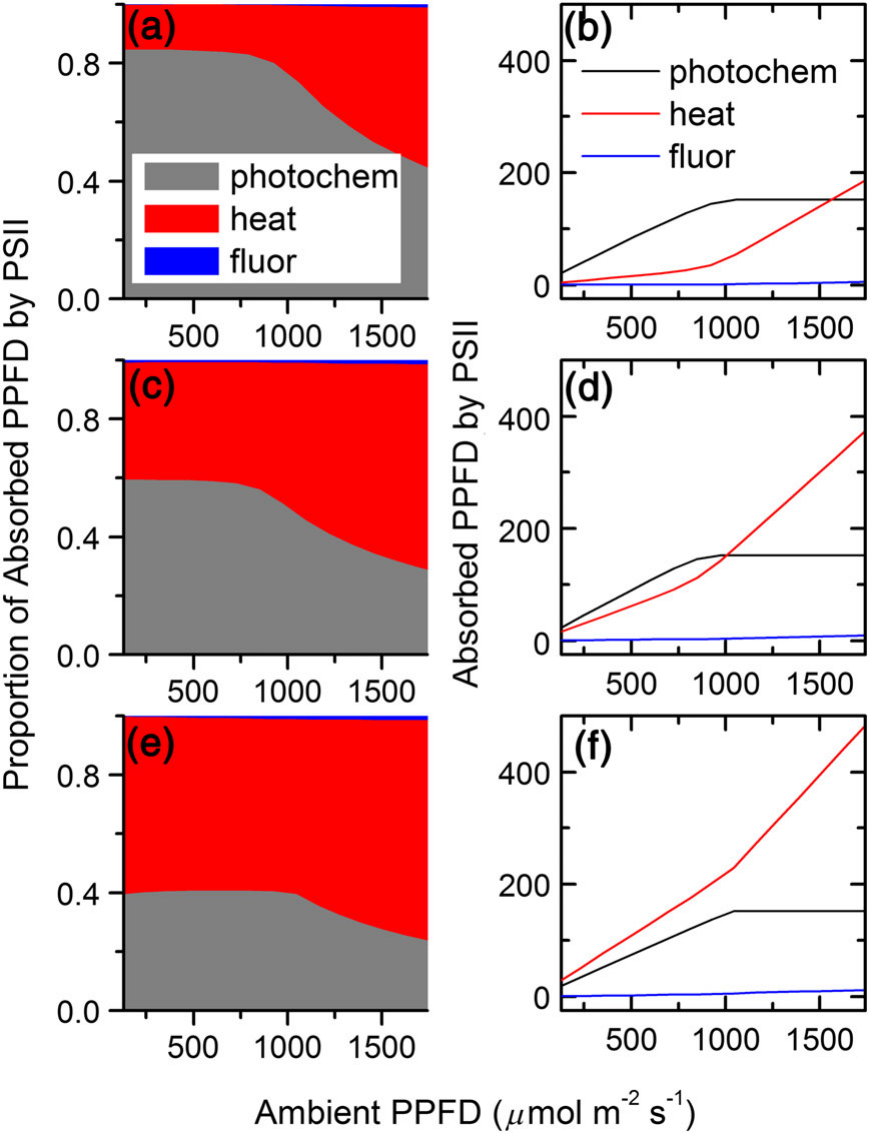
Simulated proportion (a, c, e) and amount (b, d, f) of absorbed photosynthetic photon flux density (PPFD) by photosystem II used for photo‐chemistry (grey areas or black curves), heat dissipation (red areas or red curves), and fluorescence emission (blue areas or blue curves) under different ambient PPFD and at three chlorophyll concentrations (a, b: [Chl] = 227.7 μmol m^−2^; c, d: [Chl] = 419.5 μmol m^−2^; e, f: [Chl] = 741.5 μmol m^−2^) achieved by changing antenna size of photosystems

The integrated canopy photosynthesis model scales from metabolism to canopy, which provides the capacity for studying the impacts of modification made at the molecular level on leaf and canopy level photosynthetic CO_2_ uptake rates. In this study, we explored two options of modifying leaf chlorophyll concentrations using the e‐photosynthesis model. The first one was by changing the number of photosystems units while keeping antenna size for each photosystem constant (Figure S1), and the second one was by modifying the antenna size while maintaining the number of photosystems units constant (Figure S1). For the first option, the energy conversion efficiency for one photosystem did not change because the structure of each photosystem was the same. However, for the second option, when decreasing antenna size, the proportion of absorbed PPFD used for photochemistry gradually increased and the heat dissipation and fluorescence emission gradually decreased with decreasing antenna size (Figure 3). This is because the leaf absorbance and total absorbed PPFD decreased with decreasing antenna size, but the PPFD used for photo‐chemistry was almost the same (Figure 3).

To demonstrate the impact of these two options of changing leaf chlorophyll concentration on leaf photosynthesis under different light intensities, we simulated leaf photosynthesis under different absorbed light for leaves with different chlorophyll concentrations by changing antenna size (Figure S2A) and by changing photosystems number (Figure S2B). The initial slope of the curve increased when chlorophyll concentration was decreased by changing antenna size (Figure S2A), but the initial slope decreased when chlorophyll concentration was decreased by changing photosystems number (Figure S2B). We further simulated leaf photosynthesis under different incident light. Simulation results show that when chlorophyll concentration was changed by changing antenna size, the initial slope were almost the same (Figure S2C), but the slope decreased when chlorophyll concentration was decreased by changing photosystems number (Figure S2D).

### 3.2 Distribution of PPFD in a canopy when leaf chlorophyll concentration ([Chl]) was modified

**Figure 4:**
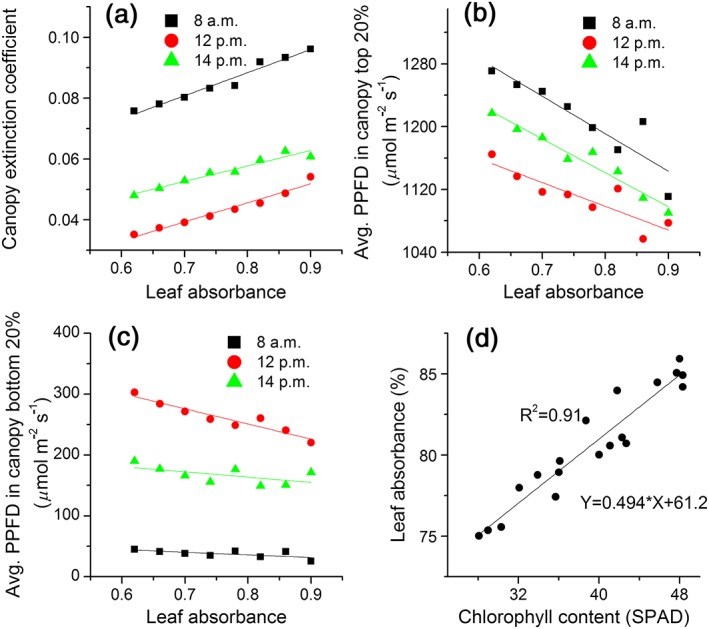
Canopy light microclimates. (a) The relationship between canopy light extinction coefficient and leaf absorbance at 8 a.m., 12 p.m., and 14 p.m. The extinction coefficient was calculated by fitting absorbed photosynthetic photon flux density (PPFD) of leaves in a canopy with the Beer's Law. (b) The PPFD in the top layer of the canopy (top 20% of leaf area index [LAI]). (c) The PPFD at the bottom layer of the canopy (bottom 20% of LAI) under different leaf absorbance. (d) The relationship between leaf absorbance and chlorophyll content as represented by the SPAD readings

Modifying leaf chlorophyll content can lead to modified light environments inside a canopy because leaf absorbance positively related to chlorophyll concentration with R‐square 0.91 (Figure 4d). We quantified light distribution in a canopy by fitting light extinction coefficient (*T*) based on the simulated PPFD in the canopy. The fitted *T* positively correlated with leaf absorbance at different times during a day (Figure 4a), allowing more light penetrating to bottom layers of the canopy under lower leaf chlorophyll concentration due to increased leaf transmittance. However, reflectance also increases with decreasing leaf chlorophyll resulting in lowered total canopy absorption. To further explore the influence of leaf chlorophyll concentration on PPFD levels within a canopy, we calculated the average PPFD in top 20% and bottom 20% of canopy heights. The PPFD was increased in both top 20% (Figure 4b) and bottom 20% (Figure 4c) canopy heights when leaf chlorophyll concentration was deceased.

### 3.3 The influence of modified leaf chlorophyll concentration on the optimal distribution of nitrogen into different components of photosystems

**Figure 5:**
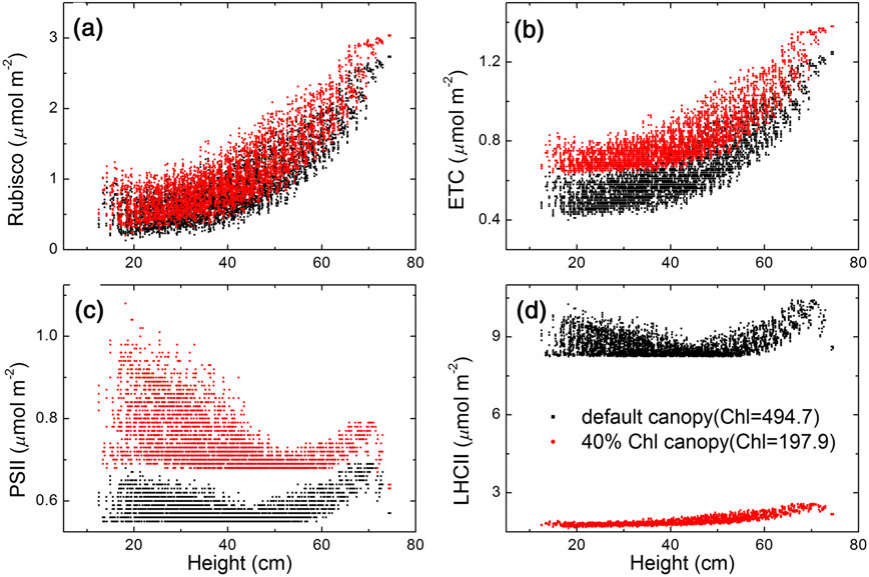
Simulated distribution of enzymes including (a) Rubisco; (b) ETC (electron transport chain components); (c) PSII (photosystem II); and (d) LHCII (light‐harvesting complex II) for a default canopy and a canopy with 40% leaf chlorophyll concentration ([Chl]). During the simulation, the total nitrogen content invested into the photosynthesis was maintained as constant. The light environment used in the simulation was for the 235th day of year in Shanghai

For a given investment of nitrogen into the photosynthetic apparatus, there needs to be an optimal allocation to maximize photosynthetic light and hence nitrogen use efficiencies. Thus, as less nitrogen is invested in the photosystems, it matters how conserved nitrogen is re‐invested. This is illustrated in two simulated scenarios. In one scenario, the antenna size was decreased without modifying content of other photosynthetic proteins, whereas in another scenario, the antenna size was decreased with increasing content of other proteins to maintain the total nitrogen invested into photosynthetic apparatus to be constant. Figure 5 illustrates these two scenarios when leaf chlorophyll concentration was decreased by 60%, though the LHC decreased dramatically, all the contents of all other enzymes, that is, Rubisco, electron transport chain (ETC), PSII in photosynthesis were increased for all leaves in the canopy (Figure 5).

### 3.4 The influence of modifying leaf chlorophyll concentration on leaf and canopy photosynthetic efficiency

**Figure 6:**
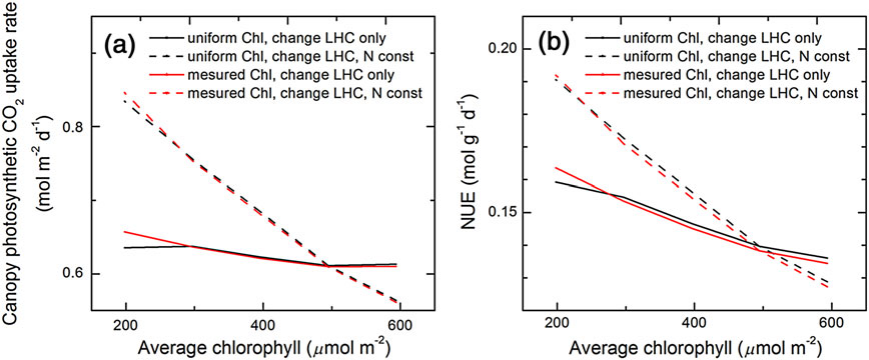
(a) Canopy photosynthetic CO_2_ uptake rate and (b) nitrogen use efficiency for canopies with different leaf chlorophyll concentrations. The chlorophyll distribution in the canopy was assumed either following a uniform distribution (black lines) or following a distribution as measured (red lines). When decreasing chlorophyll concentration, we used two different options, with one option being decreasing light‐harvesting complex (LHC) only (solid line) and the other being decreasing LHC and keeping total nitrogen in photosynthesis components constant (dashed line). The light environment used in the simulation was for the 235th day of year in Shanghai

**Figure 7:**
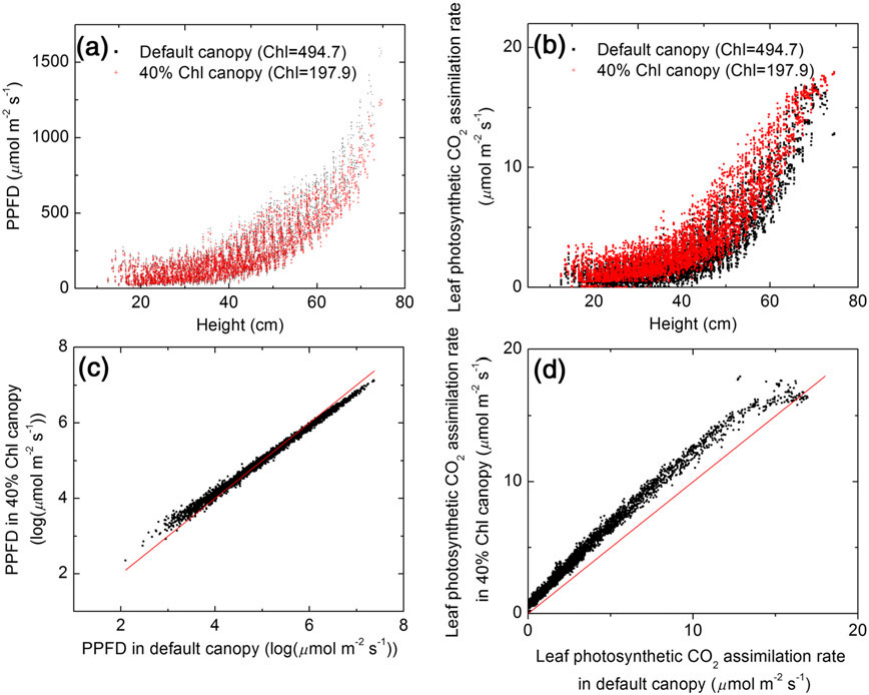
The vertical distributions of (a) absorbed PPFD and (b) leaf photosynthetic CO_2_ assimilation rate throughout the canopy for both default and 40% chlorophyll concentration ([Chl]) canopies. c and d show the comparisons of (c) absorbed PPFD and (d) photosynthetic CO_2_ assimilation rates in default and 40% [Chl] canopies. The light environment used in the simulation was for the 235^th^ day of year in Shanghai

To test the hypothesis that decreasing antenna size can improve canopy photosynthesis and nitrogen use efficiency (*NUE*), we calculated daily canopy photosynthetic CO_2_ uptake rate (*A*_*c*_) and *NUE*. Our analysis showed that the *A*_*c*_ was increased over 3% and *NUE* increased by up to 14% when leaf chlorophyll concentration decreased to 40% of its default value by reducing LHC only (Figure 6). However, when the nitrogen saved by reducing chlorophyll was reinvested to gain optimal leaf photosynthetic CO_2_ uptake, both *A*_*c*_ and NUE increased by over 30% (Figure 6). To further study the impact of decreasing chlorophyll concentration for leaves in different layers of canopy, we selected the scenario of uniform chlorophyll distribution and constant leaf N as default canopy to simulate PPFD distribution in canopy and calculate leaf photosynthesis rates for all leaves in the canopy. Decreasing the leaf chlorophyll concentration to 40% of the default canopy, we found that the light distribution in canopy was improved when decreasing chlorophyll concentration as shown in Figure 7a,c), that is, the absorbed PPFD was decreased for leaves under high PPFD but increased for leaves experiencing lower PPFD (Figure 7a,c). As the saved nitrogen from reduced chlorophyll and LHC was distributed to other enzymes in photosynthesis, leaf photosynthetic CO_2_ assimilation rate was increased for nearly all leaves in canopy (Figure 7b,d). The difference between an assumed uniform chlorophyll distribution within the canopy and measured chlorophyll distribution was minor (Figure 6).

### 3.5 The influence of modifying leaf chlorophyll concentration on canopy photosynthesis under different canopy structure features and latitudes

**Figure 8:**
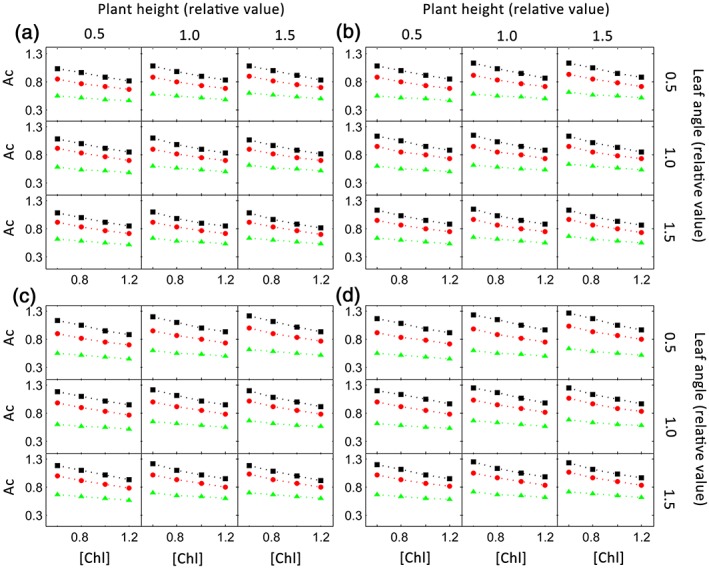
Simulated relationship between leaf chlorophyll concentration and canopy photosynthetic CO_2_ uptake rates (A_c_) for different combinations of canopy structural features, that is, plant height (plant height of the default canopy as well as 0.5 and 1.5 times of the default plant height), leaf angle (leaf angle of the default canopy as well as 0.5 and 1.5 times of the default leaf angles), and leaf area index (LAI; LAI of the default canopy [red points] as well as 0.5 times [green points] and 1.5 times [black points] of the default LAI), at four latitudes, that is, a (latitude: 45°N), b (latitude 40°N), c (latitude 31°N), and d (latitude 18°N)

Considering that modifying leaf chlorophyll concentration mainly influences leaf photosynthesis through modifying light environments inside a canopy, we studied the potential impacts of different canopy architecture and growth latitudes on the benefits of modifying leaf chlorophyll concentration on canopy photosynthesis (*A*_*c*_). Leaf angle, leaf curvature, plant height, leaf area index (*LAI*), and growth latitudes were examined in this study because they all influence the light environments inside a canopy. Our results suggest that decreasing chlorophyll concentration can always lead to an increase canopy photosynthetic CO_2_ uptake rate under these different scenarios (Figure 8) though the potential benefits of decreasing antenna size differs, for example, the benefit of decreasing leaf chlorophyll concentration was higher in canopies with higher *LAI* (Figure 8).

## 4 DISCUSSION

This paper reports development of a dynamic systems model of canopy photosynthesis and its application in exploring the potential of decreasing leaf chlorophyll concentration as a strategy to improve canopy photosynthetic CO_2_ uptake rates for canopies with a variety of architectural features and grown under different latitudes.

### 4.1 The potential of modifying leaf chlorophyll concentration on canopy photosynthesis rate

Various options have been proposed so far to increase canopy light use efficiencies, see review in Zhu et al. (2010) and recent updates in Long, Marshall‐Colon, & Zhu (2015). Among these proposed options, decreasing leaf chlorophyll content has been proposed as a viable option. The potential impacts of modifying leaf chlorophyll concentration on canopy photosynthesis has been explored earlier using a sunlit‐shaded model (Ort et al., 2011), where the leaves inside the canopy was assumed to be either sunlit or shaded (Norman, 1980). In other words, the temporal and spatial heterogeneities of light environments inside the canopy was ignored. Considering that ignoring the heterogeneity of such light environments can potentially bias the estimate of canopy photosynthetic rates, here we study the potential benefits of modifying leaf chlorophyll concentration on rice canopy photosynthetic rates. Our analysis shows that decreasing antenna size in general can increase canopy photosynthetic CO_2_ uptake rates (Figures 6 and 8), even though the magnitude of the benefit depends on both the growth latitude and a number of plant architectural parameters (Figure 8). For example, under low leaf area index, the relative benefit of decreasing antenna size will be lower (Figure 8). When the leaf chlorophyll concentration was decreased, the canopy nitrogen use efficiencies increased dramatically (Figure 6). This is due to the decreased nitrogen investment while at the same time the increased canopy photosynthetic rates (Figure 6).

The increased canopy photosynthetic CO_2_ uptake rates under decreased antenna size is attributed to two major factors. First, when the antenna size decreases, the proportion of PPFD used for photochemistry increases (Figure 3) as a result of the decreased proportion of heat dissipation (Zhu et al., 2005). This is also reflected in the increased leaf photosynthetic CO_2_ uptake rates under nonsaturated light when the antenna size was smaller (Figure S2A). Second, when the antenna size decreased, the light distribution inside the canopy was improved, in the sense that the absorbed PPFD of top leaves were slightly decreased while the absorbed PPFD of leaves at bottom layers were increased (Figure 7) due to the decreased extinction coefficient (Figure 4a). This modified light environments combined with the non‐linearity light response curve of photosynthesis (A‐Q curve) together results in a higher A_c_ (Figures 6 and 7).

If the nitrogen saved by decreasing leaf chlorophyll content can be optimally allocated to other components of photosynthesis, much higher increase in total canopy photosynthesis was predicted (Figure 6a). Our earlier study suggested that the current nitrogen investment into photosynthetic machinery is not optimal, as a result of changed global CO_2_ concentrations, which in theory can shift the control over photosynthetic CO_2_ uptake from Rubisco to RuBP regeneration (Zhu, de Sturler, & Long, 2007). This is later demonstrated in the field experiment where tobacco with overexpressed SBPase showed greater stimulation in biomass accumulation under elevated atmospheric CO_2_ concentration (Rosenthal et al., 2011). Hence, it is desirable to consider the optimal nitrogen allocation patterns together with the decreased antenna size (Zhu et al., 2007). Now the challenge is to identify the optimal option to decrease leaf chlorophyll concentration and also the antenna size. One possibility is to modify chlorophyll a oxidase, which has been reported to be related to antenna size (Masuda, Tanaka, & Melis, 2003). Another possibility is to modify FetZ, which is a major factor involved in the chloroplast division machinery and hence influence mesophyll chloroplast number (TerBush, Yoshida, & Osteryoung, 2013). In theory, decreased expression of FetZ should lead to decreased chloroplast division and hence increased leaf light transmittance, and potentially reflectance as well, due to sieve effect. The impacts of these modifications on leaf and canopy photosynthesis awaits experimental verification.

### 4.2 Potential applications of the new dynamic model of canopy photosynthesis and its future developments

Canopy photosynthesis, rather than leaf photosynthesis, should be the target to increase for higher biomass production and crop yield (Zhu et al., 2012), as has been demonstrated in cotton (Wells et al., 1986) and soybean (Harrison & Ashley, 1980). Unfortunately, the complexity of the photosynthetic process, which consists of about 100 proteins, combined with the heterogeneous microclimates, in particular light conditions inside a canopy, make it rather challenging to identify the limiting factors controlling canopy photosynthesis using the traditional transgenic approaches. The model presented here incorporates a realistic three‐dimensional plant architecture, detailed prediction of light environments inside the canopy (Song et al., 2013), together with a dynamic systems model of leaf photosynthesis (Zhu et al., 2013), which enables a direct prediction of the impacts of modifying a particular enzyme or a set of enzymes involved in photosynthesis on canopy photosynthesis and nitrogen use efficiencies of a crop with defined canopy architecture, growth location, and growth densities. Such a newly gained capacity is timely because modern biotechnologies, such as genome editing technologies (Bortesi & Fischer, 2015), now make it possible to engineer any one or combination of genes relatively easily while the challenge is to define the targets to manipulate. The model also enables evaluation of different planting strategies on canopy photosynthesis rate, as demonstrated in our recent study where we show the impact of using different planting systems, that is, even or varied row spacing, on sugarcane production (Wang et al., 2017). Skipping some rows in rice or wheat will potentially lead to decreased leaf area index, which can potentially decrease the potential benefit of decreasing antenna size. As shown in our sensitivity analysis (Figure 8), there is a benefit to canopy photosynthesis by decreasing the current chlorophyll concentration even though the magnitude of the benefit depends on both the growth latitude and a number of plant architectural parameters (Figure 8).

Though the canopy photosynthesis model presented here represents a significant advance in modelling canopy photosynthesis, there are still a number of aspects related to canopy photosynthesis simplified and hence need to be improved later. These factors can potentially influence the magnitude of the impacts of decreasing leaf chlorophyll on canopy photosynthetic CO_2_ uptake rate. First, the influence of floral structures to the light distribution inside canopies is not considered in the current study. The positions of the floral structures can differ within canopies. For example, wheat spike is usually on the top of a canopy and can shade leaves including flag leaf and the height of panicle for rice, in particular indica rice, is usually the same as or lower than that of the flag leaf. Therefore, the spikes of rice and wheat influence light canopy microenvironments differently. Most likely, the existence of floral structure decreases light levels inside canopies and hence can magnify the impacts of lower chlorophyll on canopy photosynthetic rate. Second, in the current model, the contribution of leaf sheath photosynthesis is not incorporated. Many evidences suggest that in rice and wheat, photosynthate contributed by sheath photosynthesis is important to grain filling (Guo, He, & Deng, 2013; Zhang, Zhang, Wang, & Wang, 2011) and can be 5–14% of the total final grain yield (Zhang et al., 2011). The predicted detailed light environments at different parts of a leaf sheath make it possible to calculate the contribution of sheath photosynthesis if the biochemical and physiological parameters related to sheath photosynthesis are available. Third, in the current model, the CO_2_ gradient inside the canopy is not explicitly simulated. Earlier studies have shown a moderate drawdown of CO_2_ concentration from the air immediately above the canopy to the middle of a soybean canopy at midday (Francis & Parks, 1988). Though such a drawdown only has a ~4% impact on total canopy CO_2_ uptake rate (Zhu et al., 2012); however, for canopies with much higher leaf area index in an environment with still air, the potential CO_2_ drawdown and impact on canopy photosynthesis can be greater. Therefore, future models of dynamic canopy photosynthesis also need to incorporate the dynamic changes of CO_2_ concentration inside a canopy. Under such conditions, the proportion of leaves performing light‐limited photosynthesis in a lower layer of canopies decreases. As a result, the benefit of increasing light availability for lower layer leaves will decrease. Models with explicit simulation of CO_2_ gradients inside a canopy need to be developed to quantify the impacts of decreasing leaf chlorophyll concentration on canopy photosynthesis under such cases.

## CONFLICT OF INTEREST

The authors claim no conflict of interest.

## Supporting information

Figure S1. A diagram to show the two options of changing leaf chlorophyll concentration. The first option is through changing number of photosystems and the second option is through changing the antenna size.
Figure S2. Light response curves for leaves with different chlorophyll concentrations (Chl) by changing antenna size (A, C) and by changing number of photosystems (Changing PS Number) (B, D). In A and B, absorbed light were used as X axes, while in C and D, incident light were used as X axes.
Appendix I: Determination of canopy extinction coefficient and relationship between leaf optical properties and SPAD measurements
Table S1. Leaf reflectance, transmittance and absorbance data measured for fitting the relationship between SPAD value to reflectance and transmittance.
Table S2. Abbreviations used in the paper.
Table S3. Properties of components of photosystems used in the model
