# Supplementary material for: The impact of modifying photosystem antenna size on canopy photosynthetic efficiency—Development of a new canopy photosynthesis model scaling from metabolism to canopy level processes
Source: Plant Cell Environ. 2017 Sep 21;40(12):2946–57. doi: 10.1111/pce.13041 (PMC5724688; doi:10.1111/pce.13041)
Supplement: Supplementary file 1 — Figure S1. A diagram to show the two options of changing leaf chlorophyll concentration. The first option is through changing number of photosystems and the second option is through changing the antenna size. Figure S2. Light response curves for leaves with different chlorophyll concentrations (Chl) by changing antenna size (A, C) and by changing number of photosystems (Changing PS Number) (B, D). In A and B, absorbed light were used as X axes, while in C and D, incident light were used as X axes. Appendix I: Determination of canopy extinction coefficient and relationship between leaf optical properties and SPAD measurements Table S1. Leaf reflectance, transmittance and absorbance data measured for fitting the relationship between SPAD value to reflectance and transmittance. Table S2. Abbreviations used in the paper. Table S3. Properties of components of photosystems used in the model [file PCE-40-2946-s001.doc]

**Appendix I**

**Curve fitting equations:**

**i. Canopy extinction coefficient**

Canopy extinction coefficient was calculated by fitting simulated PFPD in a canopy with Beer’s Law (Monsi and Saeki, 2005). *I_0_* is the PPFD on top of a canopy, *T* is extinction coefficient and *d* is depth in canopy.

$I=I_{0}*e^{-T*d}$ (Eqn S1)

**ii. Relationship between leaf transmittance, reflectance and SPAD value**

As chlorophyll concentration positive related to leaf absorbance, with which chlorophyll concentration influenced photosynthetic photon flux density (PPFD) distribution in a canopy, leaf chlorophyll content through canopy was measured using chlorophyll meter SPAD-502Plus and the SPAD value was calibrated to chlorophyll concentration measured with spectrophotometer. The relationship of leaf transmittance (*t*), reflectance (*r*) and SPAD value (*SPAD*) was fitted and the following equations were generated.

$Chl=47.69\cdot e^{0.0518\cdot SPAD}$ (Eqn S2)

$t=2264\cdot e^{-0.2413\cdot SPAD}+20.41\cdot e^{-0.03072\cdot SPAD}$ (Eqn S3)

$r=-0.1955\cdot SPAD+20.14$ (Eqn S4)

**Supplementary Figures:**

**
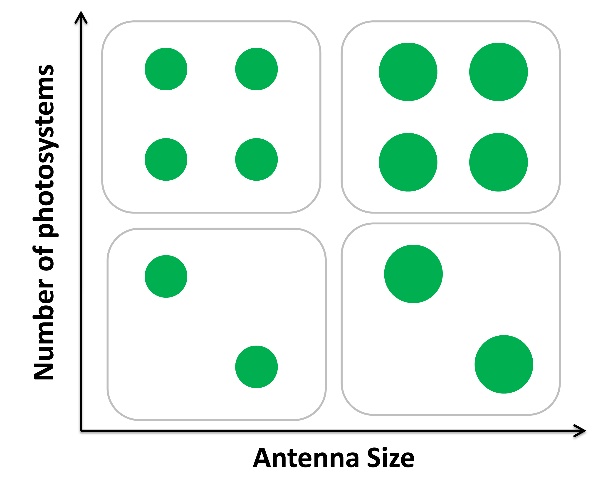
**

Figure S1. A diagram to show the two options of changing leaf chlorophyll concentration. The first option is through changing number of photosystems and the second option is through changing the antenna size.


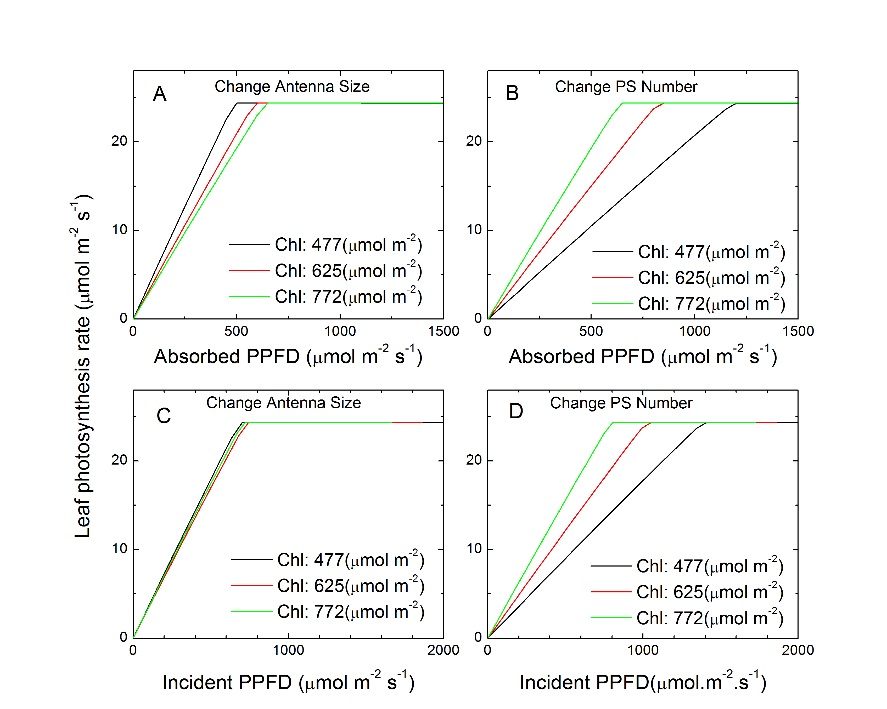


Figure S2. Light response curves for leaves with different chlorophyll concentrations (Chl) by changing antenna size (A, C) and by changing number of photosystems (Changing PS Number) (B, D). In A and B, absorbed light were used as X axes, while in C and D, incident light were used as X axes.

**Supplementary Tables:**

**Table S1**. Leaf reflectance, transmittance and absorbance data measured for fitting the relationship between SPAD value to reflectance and transmittance.

| **Genotype** | **[Chl]SPAD value** | **Reflectance** | **Transmittance** | **Absorbance** |
| --- | --- | --- | --- | --- |
| **WT** | 45.8 | 10.464 | 5.0661 | 84.47 |
| **WT** | 41.8 | 10.42 | 5.6062 | 83.974 |
| **WT** | 48.3 | 10.699 | 4.3942 | 84.907 |
| **WT** | 36.5 | 10.337 | 6.4295 | 83.233 |
| **WT** | 47.7 | 10.008 | 4.9279 | 85.064 |
| **WT** | 48.3 | 11.361 | 4.4508 | 84.188 |
| **WT** | 38.7 | 11.68 | 6.198 | 82.123 |
| **WT** | 48 | 9.8952 | 4.1823 | 85.922 |
| **Y** | 33.9 | 13.397 | 7.8231 | 78.78 |
| **Y** | 30.3 | 12.826 | 11.622 | 75.552 |
| **Y** | 42.3 | 12.256 | 6.6596 | 81.084 |
| **Y** | 36.1 | 13.849 | 6.5294 | 79.622 |
| **Y** | 32.1 | 14.61 | 7.4115 | 77.978 |
| **Y** | 42.7 | 13.093 | 6.191 | 80.716 |
| **Y** | 28.1 | 13.908 | 11.076 | 75.015 |
| **Y** | 36 | 13.73 | 7.3406 | 78.929 |
| **Y** | 41.1 | 13.195 | 6.2244 | 80.581 |
| **Y** | 29 | 14.918 | 9.7117 | 75.37 |
| **Y** | 40 | 14.103 | 5.8751 | 80.022 |
| **Y** | 35.7 | 15.06 | 7.5196 | 77.42 |

**Table S2**, Abbraviations.

| **Abbraviation** | **unit** | **Defination** | **Values used in this study** |
| --- | --- | --- | --- |
| a_1_ | m^-2^ s^-1^ | Parameters of photo-acclimation, slope of (P_max_~Rubisco) | 7.150 |
| a_2_ | m^-2^ s^-1^ | Parameters of photo-acclimation, slope of (P_max_~CE) | 14.340 |
| a_3_ | m^-2^ s^-1^ | Parameters of photo-acclimation, slope of (P_max_~ETCF) | 22.12 |
| a_4_ | m^-2^ s^-1^  μmol^-1^ | Parameters of photo-acclimation, slope of (*φ*~PSII) | 0.027 |
| b_1_ | μmol m^-2^ s^-1^ | Parameters of photo-acclimation, intersection of (P_max_~Rubisco) | 0.180 |
| b_2_ | μmol m^-2^ s^-1^ | Parameters of photo-acclimation, intersection of (P_max_~CE) | -2.699 |
| b_3_ | μmol m^-2^ s^-1^ | Parameters of photo-acclimation, intersection of (P_max_~ETCF) | -7.979 |
| b_4_ | m^-2^ s^-1^ | Parameters of photo-acclimation, intersection of (*φ*~PSII) | 0.004 |
| b | Dimensionless | The ratio of extinction coefficient of nitrogen and light in canopy |  |
| c | μmol m^-2^ s^-1^ | Enzyme concentration |  |
| I_l_ | μmol m^-2^ s^-1^ | The PPFD at the middle of the flag leaf layer. |  |
| k_cat_ | Dimensionless | Catalytic number of enzymes |  |
| N_LA_ | g m^-2^ | Nitrogen content per leaf area |  |
| $N_{LA}^{fl}$ | g m^-2^ | Nitrogen content per leaf area in flag leaf |  |
| n_b_ | g m^-2^ | The minimal leaf nitrogen concentration in leaves |  |
| n_1_ | Dimensionless | Number of LHC unit per PSII or PSI. |  |
| P_max_ | μmol m^-2^ s^-1^ | Maximal leaf photosynthesis rate |  |
| r_I/II_ | Dimensionless | Ratio of PSI and PSII. | 1.4 |
| V_max_ | μmol m^-2^ s^-1^ | Maximal reaction catalyzed by an enzyme |  |
| [Chl] | μmol m^-2^ | Concentration of chlorophyll |  |
| [Rubisco] | μmol m^-2^ | Concentration of Rubisco |  |
| [CE] | μmol m^-2^ | Concentration of enzymes except Rubisco in Carvin Cycle |  |
| [ETCF] | μmol m^-2^ | Concentration of enzymes in electron transport and couple factor |  |
| [PSII] | μmol m^-2^ | Concentration of PSII |  |
| [PSI] | μmol m^-2^ | Concentration of PSI |  |
| α | Dimensionless | Parameter in the model to estimate b | 1.105 (Moreau et al, 2012) |
| β | Dimensionless | Parameter in the model to estimate b | 4.873 (Moreau et al, 2012) |
| φ | Dimensionless | Initial slope of light response curve |  |

**Table S3**, Properties of enzymes in different groups in photosynthesis.

| **Groups** | **Enzymes, proteins and etc.** | **Kcat (s^-1^)** | **MW (mg mmol^-1^)** | **Ratios in each group** | **MW (mg mmol^-1^ group unit)** | **N content (mol N mmol^-1^ group unit)** |
| --- | --- | --- | --- | --- | --- | --- |
| **Rubisco** | Rubisco | 16 | 588000 | - | 588000 | 6.72 |
| **CE** | PGA Kinase | 540 | 45000 | 1 | 409500 | 4.68 |
|  | GAP dehydragenase | 122 | 180000 | 0.59 |  |  |
|  | aldolase | 65 | 70000 | 0.67 |  |  |
|  | FBPase | 91.6 | 160000 | 0.14 |  |  |
|  | transketolase | 138 | 160000 | 0.54 |  |  |
|  | SBPase | 162 | 66000 | 0.11 |  |  |
|  | Ribulosebiphosphate kinase | 615 | 90000 | 0.31 |  |  |
| **ETCF** | Total cytbL, or cytbH, or cytc1(cytf) | - | 99400 | 1 | 366200 | 4.19 |
|  | ferredoxin-NADP+ reductase | 139 | 33800 | 1 |  |  |
|  | ATP synthase | 55 | 466000 | 0.5 |  |  |
| **PSIIcore** | Photosystem II |  | 291800 | - | 291800 | 3.33 |
| **PSI** | Photosystem I |  | 288900 | - | 288900 | 3.3 |
| **LHCII** | Light harvesting complex II |  | 25000 | - | 25000 | 0.29 |
| **LHCI** | Light harvesting complex I |  | 20000 | - | 20000 | 0.23 |
